# In-Hospital and Interstage Mortality After Late Norwood Procedure: Acknowledging the Risks When We Are Running Out of Time

**DOI:** 10.3390/children11101262

**Published:** 2024-10-18

**Authors:** Andreea Alina Andronache, Roberta Di Cosola, Martina Evangelista, Sara Boveri, Laura Schianchi, Alessandro Giamberti, Massimo Chessa

**Affiliations:** 1 Pediatric and Congenital Heart Disease Unit, IRCCS Policlinico San Donato, 20097 San Donato Milanese, Italy; andreea.andronache84@yahoo.com (A.A.A.); rdicosola@asst-pg23.it (R.D.C.); martina.evangelista@outlook.com (M.E.); alessandro.giamberti@grupposandonato.it (A.G.); 2 Scientific Division, IRCCS Policlinico San Donato, 20097 San Donato Milanese, Italy; sara.boveri@grupposandonato.it (S.B.); laura.schianchi@grupposandonato.it (L.S.); 3 Medical School, Vita-Salute San Raffaele University, 20132 Milan, Italy

**Keywords:** Norwood procedure, risk factors, mortality

## Abstract

Background: A Norwood procedure performed after 14 days of life is notably burdened by a high mortality. We analysed the real risk and which other factors influence the mortality in late Norwood procedures. Methods: A single-centre, retrospective review of a series of consecutive patients who underwent a surgical Norwood procedure from January 2019 until December 2023. The patients’ characteristics were considered to identify the factors associated with in-hospital and interstage mortality. Results: 35 patients were included and 71% (25) of the patients underwent the Norwood procedure after 14 days of life. The median age was 27 days (6–259 days). The in-hospital mortality was 26% (9/35) with 89% (8) of the deceased being older than 15 days at the time of the surgery. Other factors that negatively affected the outcome were a restrictive interatrial septum defect (ASD) in 66% of all patients (23), the need for mechanical ventilation in 46% (16) and systemic infection prior to surgery in 43% (15). Conclusions: Age at the time of Norwood was not associated with a higher risk of mortality, but other factors such as restrictive ASD, preoperative infection and the need for mechanical ventilation prior to surgery are even more important in predicting the short-term outcome.

## 1. Introduction

Hypoplastic left heart syndrome (HLHS) has provided congenital heart disease specialists with a great deal of debate in the last fifty years. This anomaly provides a wide spectrum of anatomical variation affecting the left side of the heart resulting in different degrees of obstruction to systemic blood flow, all of which with one common issue: the incapacity of the left ventricle to provide an adequate systemic output. After birth, survival is obtained by maintaining the patency of the ductus arteriosus (DA) and unrestrictive ASD. The children with HLHS have a mortality rate of 100% if left untreated, so efforts have been made to address this malformation.

The Norwood procedure was first described in 1983 for the treatment of the HLHS. Since then, many changes have been made in performing this type of surgery, from the BT shunt to the Sano conduit and to the hybrid procedure. Nonetheless, Norwood palliation has also been applied to other congenital heart anomalies as a bridge to biventricular repair or the first stage of univentricular palliation. Although the Norwood procedure is only the first step in a three-stage palliation toward univentricular heart, it is the one with the higher mortality and morbidity, and the time between the first stage and the second stage (the Glenn procedure) named the interstage period still carries a very high risk of mortality. Considering the physiopathology of the HLHS, the aim of the Norwood operation is to provide unobstructive systemic outflow with suitable systemic output, nonrestrictive coronary flow and adequate pulmonary flow with no restriction of the venous return [1]. From the clinical point of view, this translates into efforts of balancing the systemic and pulmonary circulation, both before and after the first stage palliation.

This initial surgery is ideally performed within the first week of life to ensure optimal outcomes [2]. However, in certain scenarios, the Norwood procedure is delayed, either due to late diagnosis or other medical complications. These delays can significantly impact the patient’s survival rates both in the hospital and during the interstage period.

Although a lot of interest has been shown in this topic and better outcomes have been achieved over the last 40 years, the postoperative and interstage mortality of these patients remains high [3]. Several studies have reported a hospital mortality between 9 and 25% [3,4,5,6,7]. The interstage mortality accounts for the other 10–20% of the survivals [8,9,10]. The management of children treated with the Norwood procedure is a privilege of developed countries as it is highly time- and resource-consuming. Given the fact that complicated congenital heart disease (CHD) cannot be managed in all countries, cooperation projects have been made in order to equally treat all children. In order for this to occur, neonates must be transferred to a tertiary health care facility able to provide comprehensive cardiac and surgical treatment, albeit delaying presentation and increasing the rate of preoperative complications. Late presentation is not a contraindication for the Norwood operation [4,10].

The purpose of this article is to explore the heightened risks associated with the late Norwood procedure, particularly focusing on in-hospital and interstage mortality. By examining the factors contributing to these increased risks, health care providers can better understand the challenges and work towards improving outcomes for this vulnerable patient population. As we navigate the complexities of treating HLHS, acknowledging and addressing these risks is crucial, especially when time is a critical factor.

## 2. Materials and Methods

Data were retrospectively collected. All patients undergoing the Norwood procedure at IRCCS Policlinico San Donato (Italy) between January 2019 and December 2023 were included in the study. The primary aim of this retrospective study was to analyse patient characteristics and to identify the factors associated with in-hospital and interstage mortality. Given that most patients were hospitalised for more than 30 days post surgery, in-hospital mortality was used as the primary outcome instead of the traditional 30-day mortality.

There were no exclusion criteria. No patient died prior to the Norwood procedure. The data for this study were obtained retrospectively from medical charts, both paper and electronic. All the survivors but one were discharged during interstage and were provided with material information about red flags interstage and a paediatric cardiologist’s contact details.

As an internal protocol, before discharge parents are educated to recognise the signs and symptoms that suggest an alarming situation: “red flags”. Information like saturation, weight and milk intake was sent once a week by the child’s provider to the contact from our clinic even if the patients were to continue their follow-up visits in their home country. One child was transferred to a hospital in his home country and died interstage.

### Statistical Analysis

Numerical variables are reported as means ± standard deviation and medians and interquartile ranges (IQRs), while categorical variables are reported as counts and percentages. The normality of continuous variables was assessed using visual inspection of a qq-plot and the Shapiro–Wilk test.

Overall survival (OS) was defined as the time from the Norwood procedure to death. Patients without an event at the last follow-up were entered into the survival analysis as censored.

The Kaplan–Meier survival analysis and the log-rank test were used to estimate median follow-up and cumulative recurrence rates of death.

The variables possibly associated with outcome events were assessed by univariate analysis to estimate hazard ratios, 95% confidence intervals and *p*-values with the Cox proportional hazards regression model.

A two-tailed *p*-value < 0.05 was considered significant for all statistical tests. Statistical analyses were performed using SAS version 9.4 (SAS Institute, Cary, NC, USA).

## 3. Results

**Figure 1:**
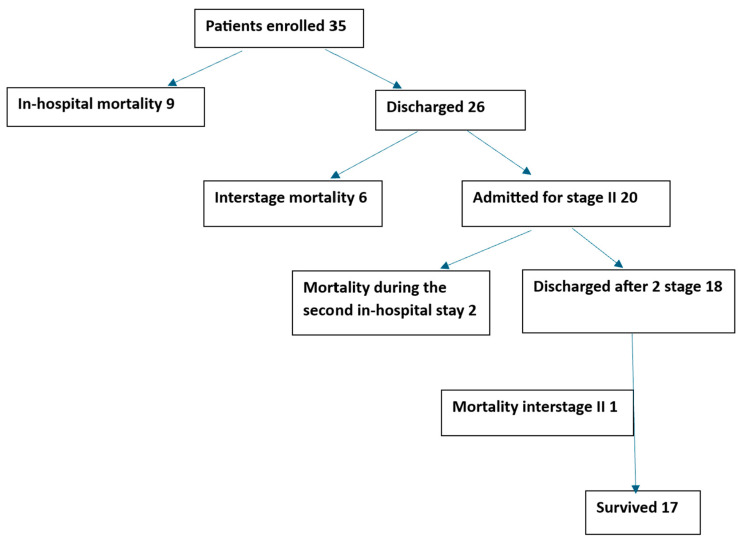
Patients included in the study.

The follow-up was completed for all patients except one—Figure 1—who was lost to follow-up after being declared unsuitable for the Glenn procedure (this was the oldest child in our study who had a Norwood–Sano operation at the age of 250 days). She survived after the procedure but due to pulmonary hypertension the Glenn procedure was not performed.

Among the 35 patients, 25 were foreigners transferred from abroad and returned to their home countries after discharge. Patient characteristics are summarised in Table 1a,b and Table 2 displays the conditions of the children at their arrival in our hospital.

We took into consideration morphological characteristics such as restrictive ASD in case of HLHS, dominant ventricle function, atrioventricular function, intubation and infection prior to surgery. We do not adopt a hybrid approach to HLHS in our centre and we prefer the Sano modification. Only one child had a Norwood–BT shunt for complex CHD (tricuspid atresia (TA) with ventricular septal defect (VSD), transposition of the great arteries (TGA) and coarctation of the aorta).

All the children (9, 25.7%) born in Italy have had a prenatal or soon-after-birth diagnosis and were transferred to our centre within the first 24 h of life. The children that came from abroad (26, 74.3%) were transferred by plane according to cooperation projects between our hospital and their countries of origin, such as Romania and Tunisia. After discharge, they returned to their home countries and continued their follow-up with local doctors until the second surgical stage. The second stage procedure and the Fontan, which is the third stage, were performed in our centre. The one child that was treated with the Norwood–Sano for aortic hypoplasia, VSD and hypoplastic aortic arch had a successful biventricular repair and he is now well and thriving.

Twenty-five (71%) of the patients underwent the Norwood procedure after 14 days of life. The median age was 27 days (range: 6–259 days).

### 3.1. Mortality

**Figure 2:**
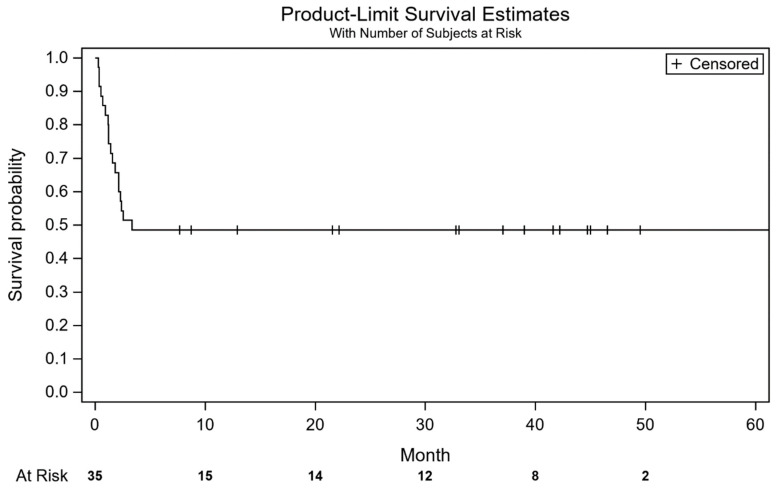
Kaplan–Meier overall (median: 3.33 (1.83—No estimate)). All the events are in the first 3.33 months.

The in-hospital mortality was 26% (9/35) with 89% (15) of the deceased being older than 15 days at the time of the surgery. Eight of the deceased children had HLHS and one other CHD. Median follow-up time was 3.2 months (CI 95%: 1.38–3.7) Figure 2. When we analysed the possible factors that influenced survival rates, we found out that age itself at the time of surgery was not statistically significant and the only statistically significant value resulted from intubation prior to surgery (*p* < 0.05), with the use of extracorporeal membrane oxygenation (ECMO) being borderline (*p* = 0.05), see Table 3. Six patients (17.1%) died during the interstage period, and all had at least one red flag that began in the days prior to admission to the hospital (saturation under 75%, fever, respiratory distress, failure to gain weight, insufficient liquid intake), see Table 4. The other two patients were transferred to our hospital in critical conditions (one with restrictive ASD and severe cyanosis and one with severe coarctation, conduit stenosis, ventricular dysfunction and ascites). In the first case, the Glenn procedure with atrial septostomy was performed but, due to persistent pulmonary hypertension and Glenn malfunction, a take-down Glenn procedure was necessary, and a BT shunt was placed.

The second patient had a reconstruction of the aortic arch and conduit replacement. He needed ECMO support, but he was successfully weaned off ECMO afterwards. Both children remained in hospital after the second procedure and died from sepsis complication and hemodynamical instability. The mortality reached a plateau after the Glenn operation and only one child died after the Glenn procedure, during the second interstage, due to non-cardiac problems (untreated pneumonia). The median of patients experienced the event of death at 3.3 months (CI 95% 1.8—no estimate).

### 3.2. Morbidity

The results of the multivariable analysis outcomes are shown in Table 5 and Table 6.

ECMO support was used in nine patients (25%)—six of them died during the in-hospital stay (unable to be weaned off ECMO or died days later from complications). One child, who was successfully discharged and also received the Glenn procedure at 16 months of age, died during the second interstage period due to an unrelated infection.

Seven children (20%) needed one or more reintervention during the in-hospital period. One child had an iatrogenic tricuspid valve lesion and needed repair of the TV twice and a take-down Sano conduit and replacement with a BT shunt, but he eventually did not survive. Two children had re-coarctation (one associated with Damus–Kaye–Stansel (DKS) anastomosis stenosis and one associated with pulmonary branch stenosis) and needed reconstruction of the aortic arch and enlargement of the DKS anastomosis in the first child and stenting of the pulmonary branch in the second case. One conduit was changed due to endocarditis. Two sternal revisions were carried out for surgical wound infection. One child who was 29 days old at the time of the surgery and was intubated since birth was unable to be weaned off the ventilator and needed a tracheostomy. He eventually died two months after the surgery.

During the interstage period, 19 patients (54.2%) had one or more red flags and 6 of them died (23.1%) (Table 7). Patients discharged with unresolved medical issues or poor follow-up plans had higher interstage mortality and morbidity.

## 4. Discussion

Successful palliation of HLHS was first described 40 years ago [11] and substantial progress has been made not only in the surgical treatment but also in the medical management of this lesion. In 2020, the European Association for Cardio-Thoracic Surgery (EACTS) and the Association for European Paediatric and Congenital Cardiology (AEPC) published guidelines for the management of children with HLHS [2]. Despite these advancements, the Norwood procedure still carries high mortality and morbidity. Managing children with such a complex pathology implies not only expert medical skills and coordination between medical specialists, but also important financial resources as these patients usually require longer intensive care unit (ICU) and hospital stays, frequent hospitalisation, home monitoring and therapies.

A “late” Norwood procedure refers to performing the surgery beyond the optimal time window, often due to late diagnosis, delayed referral, or the need to stabilise other medical conditions. This delay can exacerbate the already high risks associated with the surgery, affecting both immediate and long-term outcomes. No clear “deadline” for the Norwood procedure has been proven so multiple risk factors have to be evaluated when deciding on the approach [1]. In our study, we did not find a cut-off age as a contraindication to surgery, but we know that delaying surgery in a critically ill neonate that requires invasive monitoring will raise the risk of infections that will complicate the course of recovery [3,12,13]. Furthermore, balancing the Qp:Qs of a neonate with single-ventricle physiology can be extremely difficult, requiring intubation and invasive ventilation that could alter the highly fragile hemodynamic of these children [14,15]. Salmes-Dolzer et al. found 20 days to be an independent risk factor for the Norwood procedure in their cohort and justified this with longer periods of pulmonary overcirculation and possible cardiac decompensation [4]. As was already states, our cohort is peculiar as most of our patients come from abroad and organising the transfer increases the age at the date of surgery. Additionally, the interstage follow-up of these patients is conducted in centres without extensive experience in treating Norwood patients.

Another consideration is the fact that we do not perform the hybrid procedure in our centre, so all candidates had the Norwood procedure, despite the known risk factors (low birth weight, pre-existing comorbidities and different syndromes) [3,12,13]. ECMO is a known risk factor for in-hospital mortality [10], although in our cohort it remained borderline significant (*p* = 0.05). However, in the last two years, we have become more selective with the use of extracorporeal membrane oxygenation for patients with univentricular hearts, due to the high mortality of these patients (of the nine patients in ECMO support in our cohort, six died in-hospital).

The interstage period remains high-risk for morbidity and mortality despite the introduction of a home monitoring system [8]. Educating parents to recognise “red flags” and providing them with a portable pulse oximeter and a weight scale have increased the early detection of potentially adverse events. Six children from our cohort died interstage, all of whom had a viral or bacterial infection that led to worsening saturation and eventual cardiac arrest. These children died in a hospital after being admitted for treatment of their acute conditions. The presence of residual or recurrent anatomical lesions, such as systemic valve regurgitation, conduit obstruction, re-coarctation or restrictive ASD, increase the mortality risk [14]. Furthermore, simple childhood illnesses like fever, gastrointestinal disease and respiratory tract infections lead to dehydration, hypoxemia and increasing systemic vascular resistance causing acute fatal events in children with a minimal hemodynamic reserve.

Our study has several limitations: it included only a small sample size and most of the patients came from abroad, introducing multiple variables that may have influenced the results. However, we showed that age alone is not a contraindication for the Norwood procedure, so patients should not be refused surgery solely based on age. All risk factors should be considered in a multidisciplinary meeting and discussed with the family. Furthermore, the study conducted is retrospective so, since information is collected from patients’ charts, some details are bound to be missing. Even so, this study represents the basis on which a larger, multicentre, prospective study can be conducted.

Managing all risk factors can improve outcomes, from in utero transfer to dedicated centres to avoiding intubation, treating infections or decompressing the left atrium when a highly restrictive ASD exists, leading to higher survival rates. Prenatal diagnosis is crucial for the proper management of newborns with systemic outflow obstruction: delivering in a tertiary care setting that is confident in handling congenital heart disease allows for the use of a minimal amount of prostaglandin to maintain DA patency, avoiding the risk of complications such as apnoea, which often necessitates intubation. Acknowledging the importance of Qp:Qs ratio highlights that the goal is not achieving normal oxygen saturation, but rather accepting lower oxygen saturation with stable hemodynamics and unrestrictive pulmonary venous return. The ability to perform the Rashkind procedure in cases of a restrictive foramen ovale can improve the overall condition of the child with HLHS providing valuable time before transfer to a specialised centre for surgery.

Studies conducted on larger cohorts show that much of the early-mortality risk is due to unmodifiable factors [7] such as prematurity and anatomical variants. Introducing home monitoring programmes has been shown to reduce interstage mortality and improve connection with patients from remote areas but requires high compliance from both caregivers and the medical team [8,9]. In recent years, our centre implemented home monitoring based on models used by larger facilities treating similar congenital heart conditions but tailored to our resources. This programme includes caregiver education, a “red flags” protocol, weekly reports from parents to a doctor at our centre (even if the child undergoes intermediate checkups in their home country), early paediatric cardiology visits, and hospital admission for any “red flags”. An alternative solution could be in-hospital monitoring during the interstage of the children from remote areas who cannot attend all the intermediate visits in the referral centre, though this would be a costly approach.

In an era where changes are made continuously, the life expectancy and quality of life of children undergoing the Norwood procedure varies between centres and in different countries. Unfortunately, transplant is not a real option worldwide due to paucity of organ donors, leading to higher mortality rates. Technical surgical skills have improved leading to well-established patterns of the surgical management of patients in need of Norwood palliation. Still, in many cases, this is not enough to lower the morbidity and mortality rates to acceptable values. Prenatal diagnosis, perioperative care, home monitoring and follow-up in specialised centres, as well as health care provider training and education of the caregivers, have to be enhanced.

Another turning point is trying to change the “independent” risk factors and studies have been conducted in this direction: from avoiding the prematurity by discouraging planned deliveries before 39 weeks of gestational age to artificial wombs, new interventional techniques and centralising care to selected centres, thus enhancing experience.

Fifty years ago, the challenge of the congenital heart specialists was the survival of the patients. Currently, the focus has become the quality of life of the survivors. For this to improve, efforts must be made to centralise the care of children at high risk in specialised units and accept the fact that considerable resources in terms of money, persons and facilities are necessary.

## 5. Conclusions

This retrospective study underscores the importance of acknowledging and addressing the risks associated with late Norwood procedures. By improving early diagnosis and prompt referral to specialised centres, as well as adherence to home monitoring programmes and the rapid treatment of complications, health care providers can enhance survival rates for patients with HLHS. Future research should focus on developing strategies to ensure timely interventions and improve long-term outcomes.

## Figures and Tables

**Table 1:** (**a**) Patient data (N = 35). (**b**) Demographics.

**(a)**	
**Patient Characteristics**	**Mean ± Std–Median (25th–75th Percentile)**
Age at admission (days)	21.9 ± 41.6–15.0 (6.0–22.0)
Age at surgery (days)	27.0 ± 42.4–18.00 (8.0–27.0)
Birth weight (grammes)	3065.6 ± 432.5–3090.0 (2770.0–3300.0)
Weight at surgery (grammes)	3274.0 ± 579.6–3200.0 (2860.0–3700.0)
In-hospital stay (pre and post days)	44.4 ± 30.0–43.0 (21.0–60.0)
**(b)**	
**Sex (N = 35)**	**N (%)**
Males	19 (54.3)
Females	16 (45.7)
**Diagnosis (N = 35)**	
HLHS	23 (65.7)
TA + VSD + TGA	1 (2.9)
Aortic hypoplasia + VSD + interrupted aortic arch	3 (8.6)
DORV + aortic atresia + VSD + aortic coarctation	1 (2.9)
Severe aortic stenosis + borderline LV	3 (8.6)
TA + VSD + TGA + aortic coarctation	1 (2.9)
Unbalanced AVSD + aortic atresia + coronary fistula	1 (2.9)
DORV + subvalvular aortic stenosis + straddling TV + aortic coarctation	1 (2.9)
Dextrocardia, TGA, large VSD, hypoplastic aortic arch	1 (2.9)
**Types of HLHS (N = 23)**	
MS + AS	7 (30.4)
MA + AA	12 (52.1)
MS + AA	4 (17.4)
MA + AS	0 (0)
**ASD in HLHS (N = 23)**	
Unrestrictive	4 (17.4)
Restrictive	19 (82.6)
**Dominant ventricle’s function (N = 35)**	
Normal	24 (68.6)
Mildly reduced	5 (14.3)
Moderately reduced	5 (14.3)
Severely reduced	1 (2.9)
**Tricuspid valve regurgitation (N = 35)**	
None	9 (25.7)
Mild	15 (42.9)
Moderate	8 (22.9)
Severe	3 (8.6)
**Type of surgery (N = 35)**	
Norwood–Sano	34 (97.1)
Norwood–BT shunt	1 (2.9)

Abbreviations: TA—tricuspid atresia, VSD—ventricular septal defect, ASD—atrial septal defect, TGA—transposition of the great arteries, DORV—double-outlet right ventricle, AVSD—atrioventricular septal defect, MS—mitral stenosis, AS—aortic stenosis, MA—mitral atresia, AA—aortic atresia.

**Table 2:** Extracardiac medical conditions.

Intubation prior to admission	16 (45.7)
Infection prior to surgery	15 (42.9)
Other medical issues	
None	29 (82.9)
Gastrointestinal	1 (2.9)
Renal	2 (5.7)
Neurological	1 (2.9)
Syndromic	2 (5.7)
Arrhythmias	1 (2.9)

**Table 3:** Cox proportional hazards model (event: death).

	HR (CI 95%)	*p*-Value
Age at surgery	0.99 (0.98–1.00)	0.21
Bacterial infection	0.82 (0.32–2.07)	0.67
Intubation prior to surgery day	2.52 (1.02–6.24)	0.046
ASD in HLHS (restrictive vs. unrestrictive)	0.70 (0.24–2.04)	0.51
Age at surgery for HLHS	0.99 (0.98–1.00)	0.16
Reintervention	0.93 (0.37–2.33)	0.88
ECMO	2.38 (0.99–5.75)	0.05
Age ≥ 15	1.06 (0.28–4.09)	0.93
Age ≥ 15 + infection	1.29 (0.42–4.06)	0.66
Age ≥ 15 + Intubation	2.27 (0.92–5.54)	0.07
Age ≥ 15 + ASD in HLHS	0.82 (0.32–2.07)	0.67
Age ≥ 15 + ECMO	1.73 (0.60–4.95)	0.31

**Table 4:** Red flags.

Red Flags Interstage	Number of Patients
Oxygen saturation below 75% or above 90%	10
Respiratory distress	4
Liquid intake less than 100 mL/kg/day	5
Vomit/diarrhoea	3
Fever	6
Inconsolable crying	3
Weight loss in one day > 30 g	1
Weight gain in one day less than 20 g in 3 days	4

**Table 5:** Postoperative events.

	Median (CI 95%)
Follow-up (months)	3.20 (1.77–3.70)
	N (%)
Re-intervention	7 (20.0)
Sternal wound revision	2 (5.7)
TV plasty	1 (2.9)
mBT shunt	1 (2.9)
stent RPA + de-coarctation	1 (2.9)
Reconstruction of the ascending aorta	1 (2.9)
RV-PA conduit change	1 (2.9)
Diaphragmatic plasty	1 (2.89)
Tracheostomy	1 (2.9)
ECMO	9 (25.7)

Abbreviations: TV—tricuspid valve, RPA—right pulmonary artery, RV-PA—right ventricle to pulmonary artery.

**Table 6:** In-hospital complications in non-survivors (N = 9).

Complications	Patients
Arrhythmia	2
Sepsis	6
Arterial thrombosis	1
Neurological problems	1
TV iatrogenic lesion	1
Myocardial infarction	1
LCOS	2

Abbreviations: TV—tricuspid valve, LCOS—low cardiac output syndrome.

**Table 7:** Interstage complications.

Interstage Re-Admission to Hospital	9 (34.6)
Death	6 (23.1)
Infection	7 (20.0)
Restrictive ASD	1 (3.9)
Re-coarctation	2 (7.7)
RV-PA conduit stenosis	1 (3.9)
Hepatic cavernoma	1 (3.9)
Endocarditis	1 (3.9)

## Data Availability

Data are available from the corresponding author upon reasonable request. The data are not publicly available due to privacy reasons.
